# Supplementary material for: Caveolae-Associated Protein 3 (Cavin-3) Influences Adipogenesis via TACE-Mediated Pref-1 Shedding
Source: Int J Mol Sci. 2020 Jul 15;21(14):5000. doi: 10.3390/ijms21145000 (PMC7404391; doi:10.3390/ijms21145000)
Supplement: Supplementary file 1 [file ijms-21-05000-s001.pdf]

Supplementary Information

Caveolae-Associated Protein 3 (Cavin-3) Influences Adipogenesis via TACE-Mediated Pref-1 Shedding

Phil June Park[1,*], Sung Tae Kim[2,*]

Supplemental Table 1. Primer list

Gene Symbol	Detector	Gene Name
Cavin-3/SRBC /PRKCDBP	Mm00466330_g1	Cavin-3/serum deprivation response factor-related gene product that binds c-kinase/protein kinase C delta binding protein
Dlk1 (Pref-1)	Mm00494477_m1	delta-like 1 homolog (preadipocyte factor 1)
PPARγ	Mm01184322_m1	peroxisome proliferator-activated receptor gamma
Adipoq	Mm00456425_m1	adiponectin
TACE (Adam17)	Mm00456428_m1	TNFα-converting enzyme (Adisintegrin and metallopeptidase domain 17)
FAS	Mm00662319_m1	fatty acid synthase
SCD-1	Mm00772290_m1	stearoyl-Coenzyme A desaturase 1
aP2	Mm00433188_m1	adipocyte fatty acid binding protein 2
CEBPB	Mm00843434_s1	CCAAT/enhancer-binding protein beta (C/EBPβ)
CEBPD	Mm00786711_s1	CCAAT/enhancer-binding protein delta (C/EBPδ)

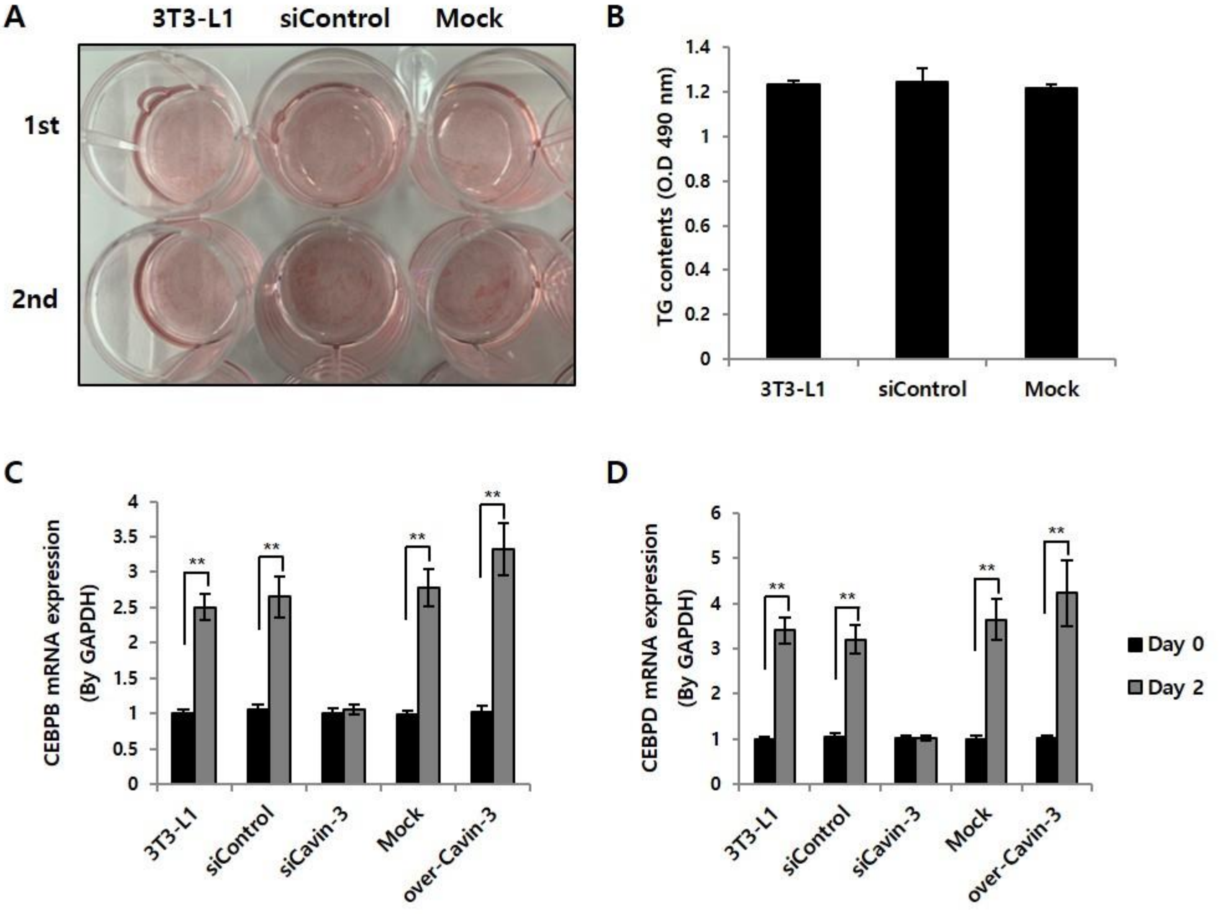

Supplementary Figure S1. The comparison of differentiation degree among control groups and the mRNA expressions level of *C/EBPβ* and *C/EBPδ* at day 2 of differentiation. (A) The ORO staining and TG content were validated in3T3-L1, siControl and Mock cells after 8 days of differentiation, respectively. (B) The TG content was quantified by Sudan II staining the cells (n = 3) by measuring the absorbance at 490 nm. The mRNA expression of (C) *C/EBPβ* (*CEBPB*) and (D) *C/EBPδ* (*CEBPD*) was determined using RT-qPCR with specific primers in 3T3-L1, siControl, siCavin-3, Mock and over-Cavin-3 cells at day 2 of differentiation. The mRNA expression of individual genes was validated in the indicated cell lines (n = 3). Data are presented as the means ± SD (*$p < 0.05$, **$p < 0.01$; data were analyzed with an unpaired Student's t-test)

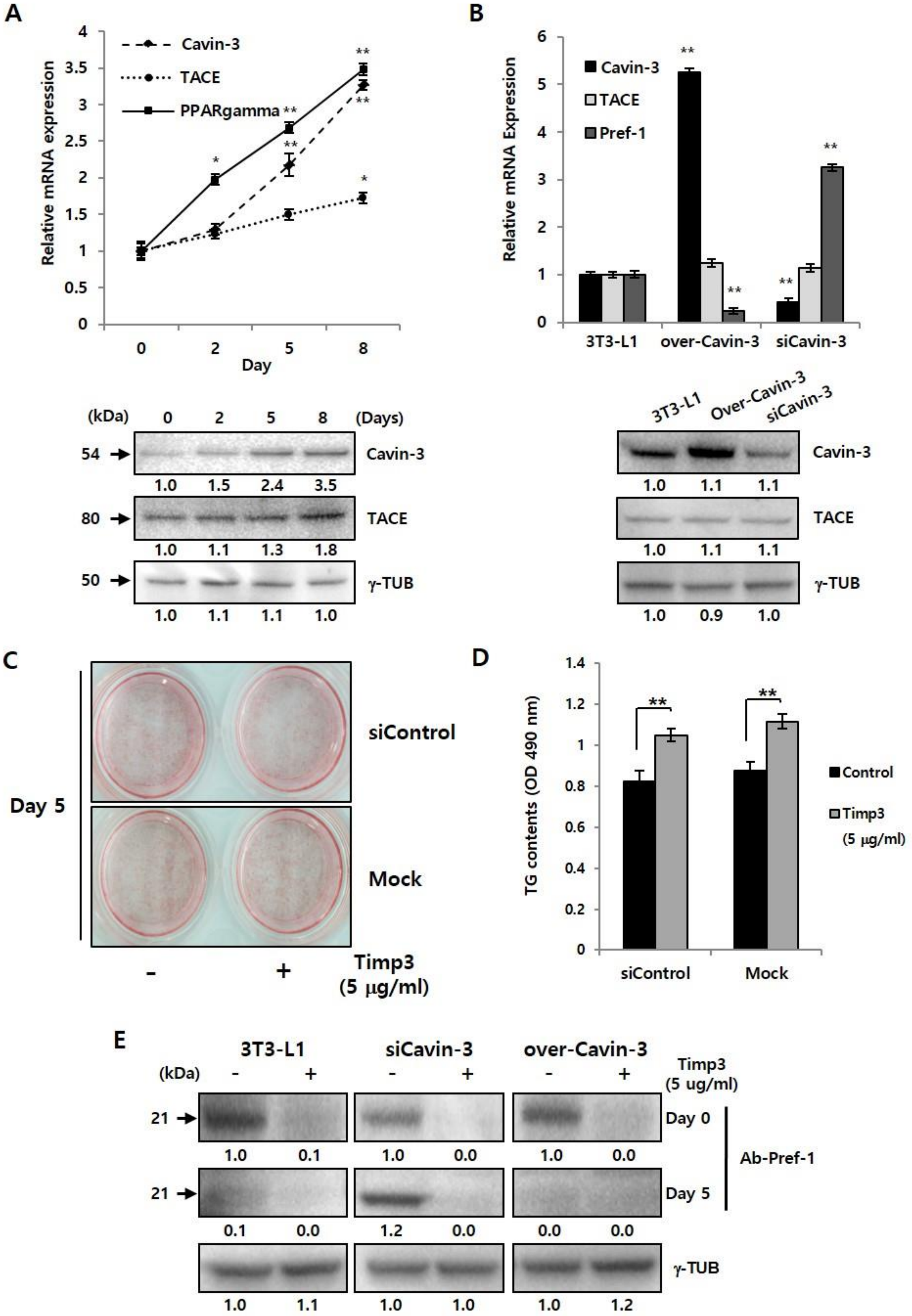

A
Cavin-3
TACE
PPARgamma
Relative mRNA expression
Day
(kDa)
(Days)
Cavin-3
TACE
γ-TUB
B
Relative mRNA Expression
Cavin-3
TACE
Pref-1
3T3-L1
over-Cavin-3
siCavin-3
Over-Cavin-3
C
Day 5
siControl
Mock
Timp3
(5 μg/ml)
D
TG contents (OD 490 nm)
Control
Timp3
(5 μg/ml)
E
3T3-L1
siCavin-3
over-Cavin-3
Timp3
(5 ug/ml)
Day 0
Day 5
Ab-Pref-1
γ-TUB

Supplementary Figure S2. The mRNA and protein expression level of *TACE* and the timp3 effect on adiogenesis and Pref-1 shedding process. (A) The Cavin-3 and TACE mRNA and protein expression were determined in 3T3-L1 adipocytes during adipogenesis, and (B) in 3T3-L1, over-Cavin-3 (n = 3) and siCavin-3 (n = 3) cells after 8 days of differentiation. The mRNA expression of individual genes was validated in the indicated cells (n = 3). Data are presented as the means ± SD. (*$p < 0.05$, **$p < 0.01$; data were analyzed with an unpaired Student's t-test) and γ-TUB was used as a control and the grouping of blots cropped from different gels. (C) The ORO staining and (D) TG content were validated in siControl and Mock cells after 5 days of differentiation, respectively. The TG content was quantified by staining the cells (n = 3) with ORO and measuring the absorbance at 490 nm. Data are presented as the means ± SD (*$p < 0.05$, **$p < 0.01$; data were analyzed with an unpaired Student's t-test). (E) Effect of timp3 on Pref-1 cleavage was analyzed by western blotting in normal 3T3-L1, siCavin-3, and over-Cavin-3 cells. Total protein was prepared before and 5 days after differentiation induction. γ-TUB was used as a control and the grouping of blots cropped from different gels.

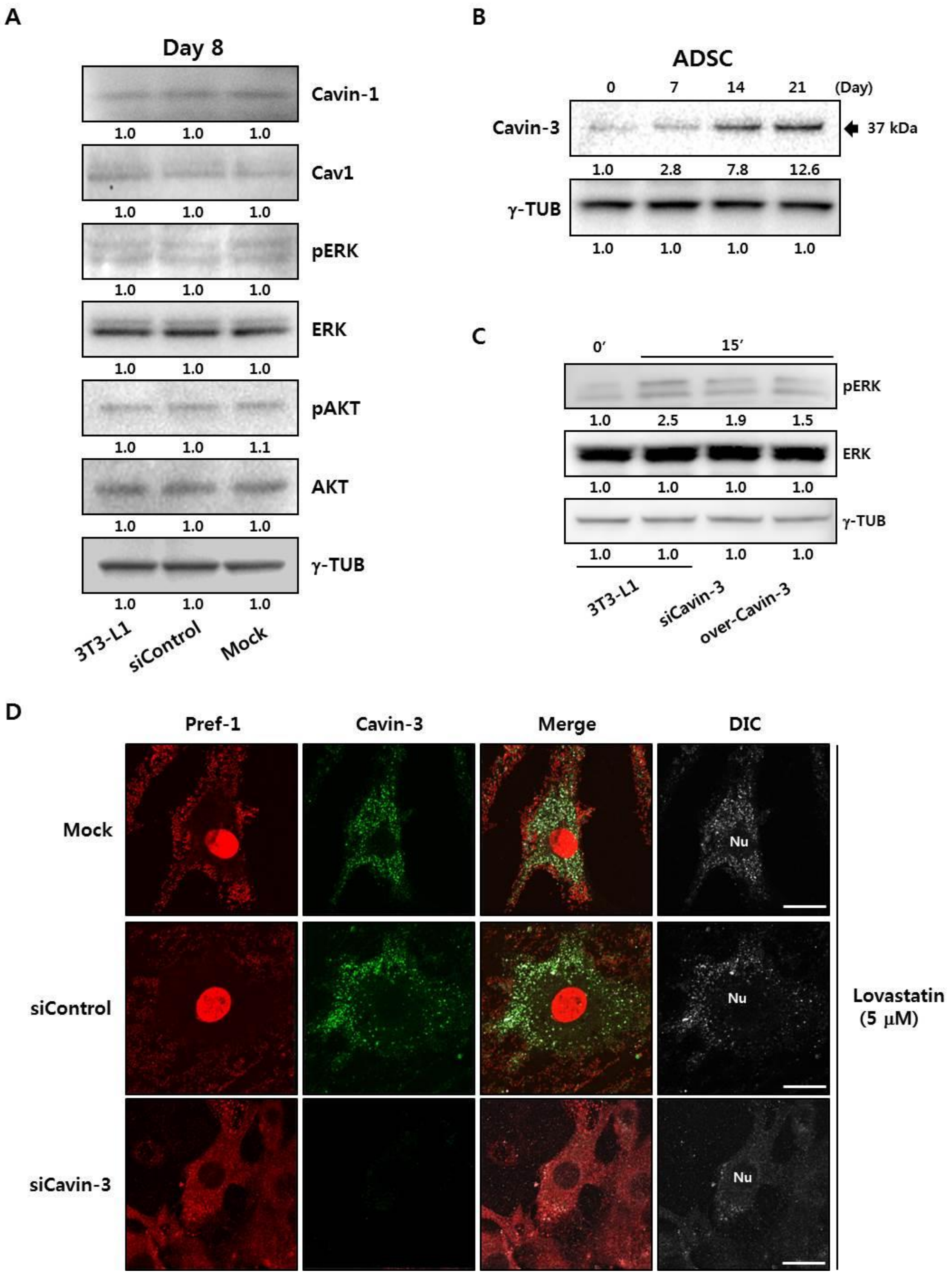

A
Day 8
Cavin-1
1.0 1.0 1.0
Cav1
1.0 1.0 1.0
pERK
1.0 1.0 1.0
ERK
1.0 1.0 1.0
pAKT
1.0 1.0 1.1
AKT
1.0 1.0 1.0
γ-TUB
1.0 1.0 1.0
3T3-L1
siControl
Mock
B
ADSC
0 7 14 21 (Day)
Cavin-3
37 kDa
1.0 2.8 7.8 12.6
γ-TUB
1.0 1.0 1.0 1.0
C
0′
15′
pERK
1.0 2.5 1.9 1.5
ERK
1.0 1.0 1.0 1.0
γ-TUB
1.0 1.0 1.0 1.0
3T3-L1
siCavin-3
over-Cavin-3
D
Pref-1
Cavin-3
Merge
DIC
Mock
siControl
siCavin-3
Nu
Lovastatin
(5 μM)

Supplementary Figure S3. The protein expression levels in various conditions at day 8 of differentiation, and subcellular localization of Cavin-3 and Pref-1 by lovastatin treatment at day 5 of differentiation. (A) The specific protein expression levels were validated among control groups, such as 3T3-L1, siControl and Mock, after 8 days of differentiation. γ-TUB was used as a control and the grouping of blots cropped from different gels. (B) The Cavin-3 protein expression levels during adipogenesis in ADSC. γ-TUB was used as a control and the grouping of blots cropped from different gels. (C) The protein expression of total ERK and phosphorylated ERK at 0 and 15 minutes after adipogensis induction. γ-TUB was used as a control and the grouping of blots cropped from different gels. (D) Mock, siControl and siCavin-3 cells were fixed with anti-Pref-1 (red) and anti-Cavin-3 (green) antibodies at day 5 of differentiation. Cells were imaged with a confocal microscope. Scale bar = 5 μm.

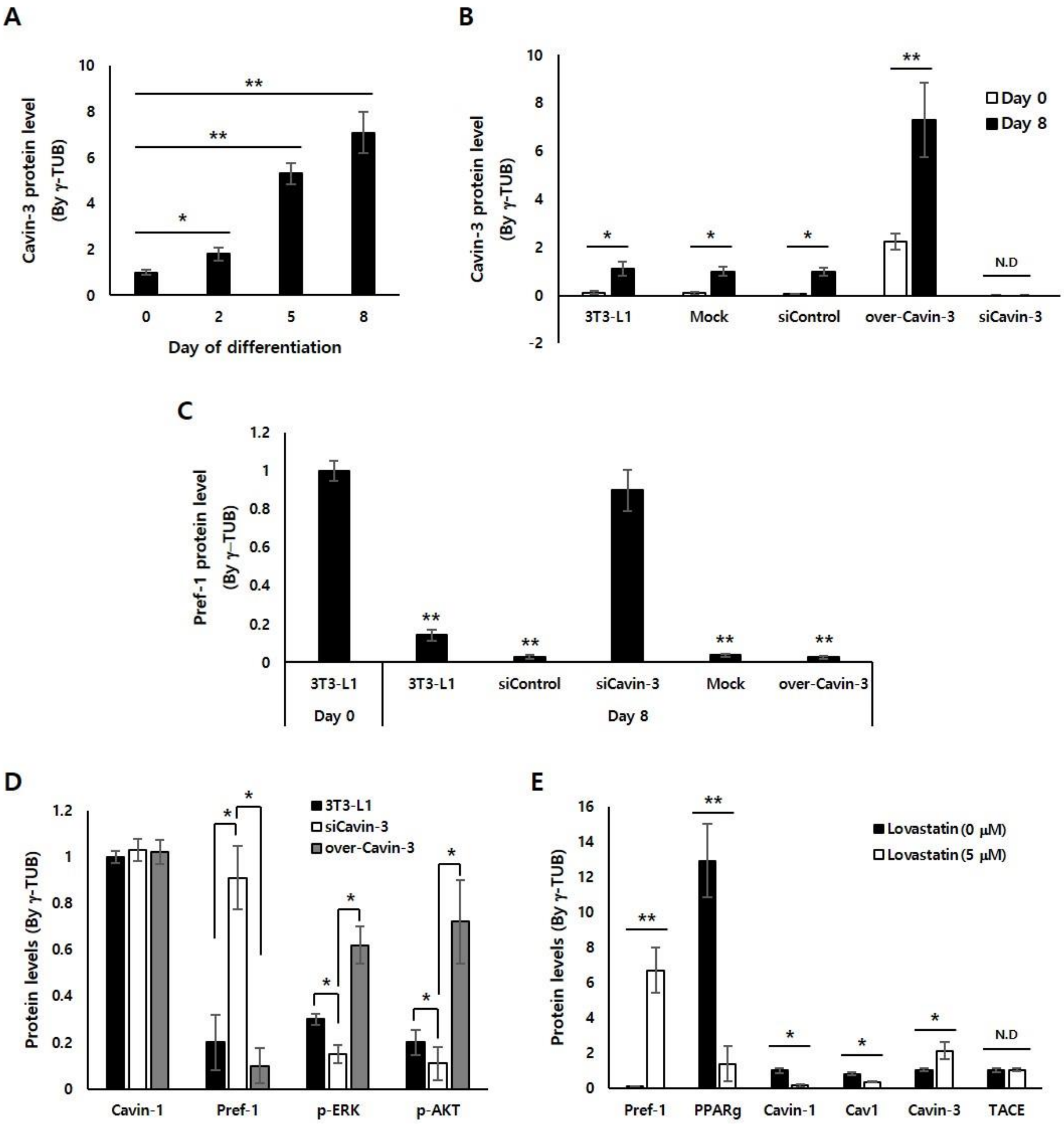
A
Cavin-3 protein level
(By γ-TUB)
10
8
6
4
2
0
**
**
*
0
2
5
8
Day of differentiation
B
Cavin-3 protein level
(By γ-TUB)
10
8
6
4
2
0
-2
□ Day 0
■ Day 8
*
*
*
**
N.D
3T3-L1
Mock
siControl
over-Cavin-3
siCavin-3
C
Pref-1 protein level
(By γ-TUB)
1.2
1
0.8
0.6
0.4
0.2
0
**
**
**
**
3T3-L1
3T3-L1
siControl
siCavin-3
Mock
over-Cavin-3
Day 0
Day 8
D
Protein levels (By γ-TUB)
1.2
1
0.8
0.6
0.4
0.2
0
■ 3T3-L1
□ siCavin-3
■ over-Cavin-3
*
*
*
*
*
*
Cavin-1
Pref-1
p-ERK
p-AKT
E
Protein levels (By γ-TUB)
16
14
12
10
8
6
4
2
0
■ Lovastatin (0 μM)
□ Lovastatin (5 μM)
**
**
*
*
*
N.D
Pref-1
PPARg
Cavin-1
Cav1
Cavin-3
TACE

Supplementary Figure S4. The quantitative analysis of protein expression levels in various conditions. (A) Cavin-3 protein level were quantified in various time points (day 0, 2, 5, and 8) during adipogenesis, and (B) compared between immature (day 0) and mature (day 8) state in various cell lines. (C) Pref-1 protein level was analyzed in various cell lines, such as normal 3T3-L1 (day 0 and day 8), siControl, Mock, siCavin-3 and over-Cavin-3 on day 8. (D) Each protein level was quantitatively analyzed in various cell lines on day 8, and (E) each data compared to each other depending on lovastatin treatment in 3T3-L1. Each quantitative level was normalized by γ-TUB. All data were presented as the mean ± SD. (*$p < 0.05$, **$p < 0.01$; data were analyzed with an unpaired Student's t-test).
